# Dental anomalies inside the cleft region in individuals with nonsyndromic cleft lip with or without cleft palate

**DOI:** 10.4317/medoral.20757

**Published:** 2015-11-30

**Authors:** Jamile Sá, Luana Araújo, Laís Guimarães, Samário Maranhão, Gabriela Lopes, Alena Medrado, Ricardo Coletta, Silvia Reis

**Affiliations:** 1 Department of Basic Science, Bahiana School of Medicine and Public Health, Salvador, Bahia, Brazil; 2 Department of Oral Diagnosis, School of Dentistry, State University of Campinas, Piracicaba, São Paulo, Brazil

## Abstract

**Background:**

Individuals with non syndromic cleft lip with or without cleft palate (NSCL±P) present high frequency of dental anomalies, which may represent complicating factors for dental treatment. The aim of this study was to investigate the prevalence of dental anomalies inside cleft area in a group of Brazilians with NSCL±P.

**Material and Methods:**

Retrospective analysis of 178 panoramic radiographs of patients aged from 12 to 45 years old and without history of tooth extraction or orthodontic treatment was performed. Association between cleft type and the prevalence of dental anomalies was assessed by chi-square test with a significance level set at *p*≤ 0.05.

**Results:**

Dental anomalies were found in 88.2% (n=157) of the patients. Tooth agenesis (47.1%), giroversion (20%) and microdontia (15.5%) were the most common anomalies. Individuals with unilateral complete cleft lip and palate (CLP, *p*<0.0001), bilateral complete CLP (*p*=0.0002) and bilateral incomplete CLP (*p*< 0.0001) were more affected by tooth agenesis than individuals with other cleft types. The maxillary lateral incisors were the most affected teeth (*p*<0.0001).

**Conclusions:**

The present study revealed a high frequency of dental anomalies inside cleft region in NSCL±P patients, and further demonstrated that patients with unilateral complete CLP and bilateral incomplete CLP were frequently more affected by dental anomalies. Moreover, our results demonstrate that dental anomalies should be considered during dental treatment planning of individuals affected by NSCL±P.

**Key words:**Nonsyndromic cleft lip with or without palate, dental anomaly, tooth agenesis, microdontia.

## Introduction

Non syndromic cleft lip with or without cleft palate (NSCL±P) is the most common orofacial birth defect, with prevalence ranging from 0.36-1.54 per 1.000 live births in Brazil (1,2). The oral clefts and the development of tooth germs have close embryological association in terms of timing and anatomical position (3), with critical events related to teeth, lip and palate formation occurring almost simultaneously (4). Dental alterations are significantly more frequent in subjects born with oral clefts if compared to the general population (5-9). In the cleft area, agenesis of the maxillary lateral incisors is the most prevalent dental anomaly, which is probably resulted of local effects of cleft (3,10,11). Supernumerary tooth are the second most common anomaly (12).

The aim of this study was to investigate the prevalence of dental anomalies inside the cleft area in a group Brazilian patients with NSCL±P.

## Material and Methods

In this retrospective cross-sectional study, 897 clinical records and panoramic radiographs of individuals with NSCL±P assisted at the Reference Center for Craniofacial Anomalies of the Santo Antonio Hospital, Salvador, Bahia, Brazil, were reviewed. Due to inability to accurately identify all dental anomalies, cases without complete dental history, with dental extraction, with previous orthodontic treatment and at an early age (<12-year old) were excluded. Patients with cleft palate only (CPO) were not included. At this point, the study ended up with 178 cases. Sub phenotypes were classified according to cleft extension - complete or incomplete, and laterality - unilateral or bilateral.

Radiographs with acceptable sharpness, contrast and density were assessed by a single calibrated examiner. The following anomalies were identified: tooth agenesis, giro version, microdontia, supernumerary tooth, included/impacted tooth, ectopic tooth, dental transposition and accessory cuspid. Statistical analysis first comprised the description of the frequencies and types of dental anomalies. Association between cleft type and the prevalence of dental anomalies was assessed by chi-square test with a significance level set at *p*≤ 0.05.

Written informed consents were obtained and the study carried out with approval of the ethics committee of the Bahiana School of Medicine and Public Health, Salvador, Bahia, Brazil.

## Results

Out of 178 patients with NSCL±P, 91 (51.1%) corresponded to males and 87 (48.9%) to females. The age of the patients ranged from 12 to 45 years old. Cleft lip and palate (CLP) was the most frequent type of oral cleft (n=135, 75.9%), particularly unilateral complete (n=90, 66.7%) and bilateral incomplete (n=34, 25.2%). The less frequent subtypes were unilateral incomplete CLP (n= 4, 2.9%) and bilateral complete CLP (n= 7, 5.2%). Cleft lip only (CLO) was found in 43 (24.1%) patients, and unilateral incomplete (n=23, 53.5%) and unilateral complete (n=15, 34.9%) were the extensions more common of CLO. In general, complete clefts (63.5%) were more frequently observed than incomplete clefts, and unilateral (74.1%) were more frequently observed than bilateral.

Dental anomalies were found in 157 patients, 35 (22.3%) with CLO and 122 (77.7%) with CLP. A single anomaly was identified in 21 individuals (11.8%) and 157 (88.2%) had multiple anomalies. Associations of agenesis and giro version (30.3%), followed by giro version and microdontia (16.0%) were the most frequent.  Table 1 describes the distribution of the dental anomalies according to cleft extension. Individuals with tooth agenesis represented 47.1% of the sample, followed by those with giro version (20%) microdontia (15.5%), impacted tooth (7.5%), supernumerary tooth (3.8%), transposition (3.4%), ectopic tooth (2.3%) and accessory cusp (0.4%). Patients with unilateral complete CLP (n=107) and bilateral incomplete CLP (n=85) were the most affected. Concerning CLO, 25 dental anomalies were observed in patients with unilateral incomplete and 18 were found in patients with unilateral complete. Agenesis was more frequent in unilateral complete FLP (*p*<0.0001) and in bilateral complete CLP (*p*=0.0002). Tooth agenesis (*p*<0.0001), giro version (*p*<0.0001) and microdontia (*p*<0.005) were significantly more frequent in CLP group than in CL group. Isolated tooth agenesis was more frequent than multiple agenesis (*p*=0.001). The most affected teeth were the lateral incisors (n=110), central incisors (n=12) and canines (n=3).

**Table 1:**
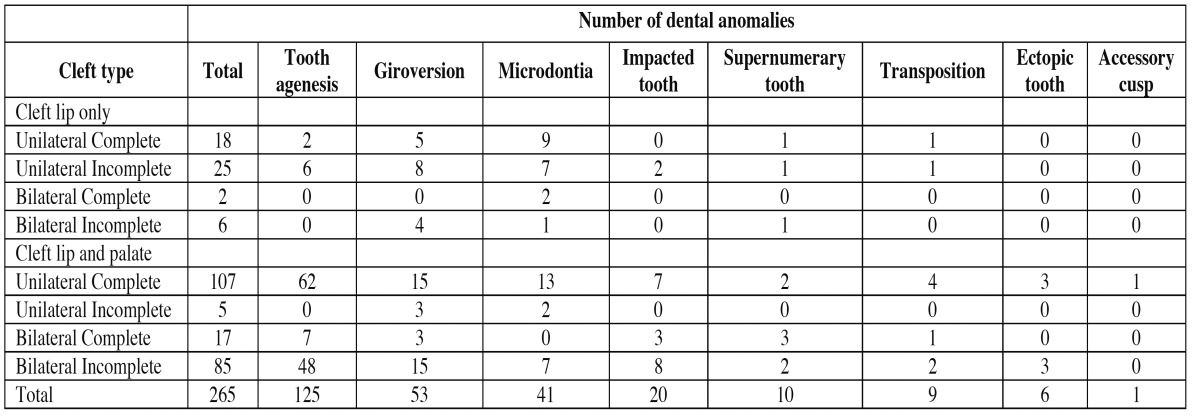
Distribution of the dental anomalies according to cleft extension.

Frequency of individuals with dental anomalies according to cleft extension is depicted in  Table 2. Individuals with unilateral complete CLP (*p*<0.0001), bilateral complete CLP (*p*=0.0002) and bilateral incomplete CLP (*p*< 0.0001) were more affected by tooth agenesis than subjects with other cleft types. Those with unilateral complete CLP were more affected by multiple anomalies (*p*=0.016) and individuals with unilateral complete CL were more affected by microdontia (*p*<0.0001).

**Table 2:**
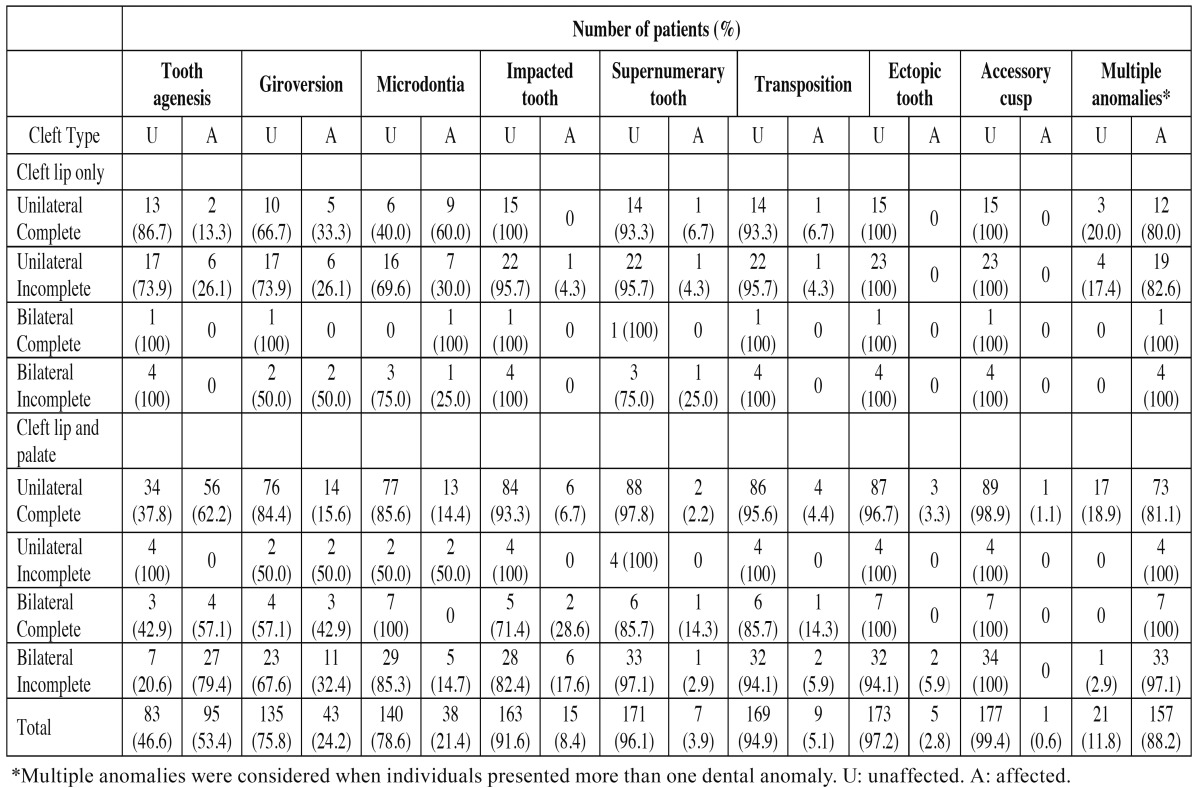
Frequency of individuals with dental anomalies according to cleft extension.

## Discussion

Oral clefts may present variable severity degree according to its extension, causing various anatomical and functional alterations (13,14). In this study we investigated the prevalence of dental anomalies inside cleft area (between incisors and canines) in different extensions of the cleft. Out of the total, 35.7% of the patients presented multiple dental anomalies. The greatest frequency of agenesis was associated to giro version (30.3%) followed by giro version and microdontia (16.0%). Such associations have not been listed in literature. It is only found that microdontia outside affected area is associated to agenesis inside the cleft (7).

According to literature, the number of affected teeth increases with severity of the cleft phenotype (5,6,12,15,16). The most extensive cleft type is bilateral complete CLP. The cleft breaches the maxilla in all its extension, from the lip to the uvula. We expected to find in this phenotype, a greater frequency of multiple dental anomalies and a greater number of affected teeth. However, our results revealed that patients with unilateral complete CLP were more affected by multiple dental anomalies (*p*= 0.016) than those with other types of cleft were. Regarding the number of affected teeth, the greatest rates were also found in unilateral complete CLP (n=114) if compared to bilateral complete CLP (n=17). Although the sample of individuals with bilateral complete CLP is small, only seven patients such results suggest that disturbed dental development does not necessarily increase with the cleft severity. In Brazilian population a study showed that patients with CLP have the highest rates of multiple dental anomalies out of the cleft area, if compared to other sub phenotypes (7).

The frequency of tooth agenesis, both in and outside the cleft region, is significantly increased in persons with clefts compared with the control population (6,7,9,17,18). Multiple factors have been suggested to justify dental agenesis close to affected area. The osseous defect caused by the cleft (10), congenital or surgery-caused low blood supply, or even low ectomesenchymal supply (12,19). Our results revealed agenesis in 45.9% of cleft patients and was more frequent in unilateral complete CLP (*p*<0.0001), CLP bilateral complete (*p*=0.0002) and bilateral incomplete CLP (*p*<0.0001). Stahl *et al*. 2006, also found prevalence of agenesis in 46.6% of European cleft individuals. In literature, we found high frequency of agenesis in bilateral or unilateral clefts of lip, alveolus, and palate (52.6% and 52.6%) (3,12). Our research pointed a total of 125 missing teeth from which the maxillary lateral incisor was the most affected one (*p*<0.0001), found in the most aggressive cleft phenotypes. These findings are explained by the anatomic proximity of lateral incisor to the cleft (7,8,10,20). Outside the cleft area, lateral incisors are also the most affected teeth (5,8,16), and more frequent in individuals with unilateral complete CLP (7).

Researches about the prevalence of giro version in cleft individuals are scarce. Our results show that giro version was associated to CLP (*p*=0.005) group, and more frequent in individuals with unilateral incomplete CLP (50.0%) and bilateral complete (42.9%). Tortora *et al*. (17) have showed that giro version occurred in lateral and central incisors, in or outside the cleft, in individuals with unilateral or bilateral CLP. Other studies reported giro version in anterosuperior area, though without association with the cleft type. (21,22).

Microdontia is reported as a partial expression of the same genetic flaw that defines agenesis (23). According to Werner and Harris (24) tooth size in unilateral CLP patients is significantly smaller than in noncleft individuals, suggesting that the compromised growth potential of CLP patients may affect tooth development. In our study, microdontia has been found in 18.4% of the sample and it was more frequent in unilateral complete CL, (*p*< 0.0001). Outside the cleft area, the prevalence of microdontia has greater rates and reach 48.2% (25,26). In a group of Brazilian patients with NSCL/P, the frequency was significantly higher than those observed in general population (9,18).

Impacted teeth were observed in 7.2% of the sample with higher frequency in individuals with bilateral complete (28.6%) and incomplete (17.6%) CLP. In the Brazilian population, this anomaly has been reported outside the cleft area in 8.8% of the individuals, (9). Ackan *et al*. (21) showed frequencies of impacted teeth varying from 1.9 to 29.2%, in the anterior region and premolars in CLP groups, with higher rates on the cleft side. Dental transposition is an uncommon dental alteration with a genetic origin that has been associated with other severe dental anomalies (27,28). Outside cleft area, such anomaly has been associated with tooth agenesis (18). In our study, dental transposition reached 4.3% and was more associated with bilateral complete CLP (14.3%) and incomplete CLP (5.9%). Wu *et al*. (26) also described dental transpositions associated with complete CLP, but in lower percentage than observed in our findings (10.6%). Supernumerary teeth were observed in only 3.4% of the sample. Kim and Baek (19) found 5.4% and Tortora *et al*. (17) 7.3%. Other studies related the presence of supernumerary teeth in the cleft area as the second most common dental anomaly (12,29). Outside cleft area, supernumerary teeth were quite often associated with unilateral complete CLP (7). Our results showed that 25% of the individuals with bilateral incomplete CL and 14.3% with bilateral complete CLP had supernumerary teeth. Wu *et al*. (26) reported 15% of prevalence among patients with CL without commitment of the alveolar edge and 13.2% in individuals with bilateral CLP. There are two hypothesis about supernumerary teeth occurrence in cleft patients. The first suggests that the odontogenic region of the lateral incisor comes from the medial nasal and maxillary processes, and the non fusion of these two processes results in two separated lateral incisors (12). Another hypothesis is that supernumerary teeth come from the post fusion rupture of the cleft in the lateral incisor area, and the tooth germ of the lateral incisor is split in two separate teeth (26).

Ectopic teeth were found in 2.4% of the sample and were registered in 3.3% of the patients with unilateral complete CLP and 5.9% of individuals with bilateral incomplete CLP. Ectopic teeth prevalence has not been documented at anterior portion of maxillary cleft patients. Out of the cleft zone, this anomaly is considered more prevalent in NSCL±P patients than those observed in the general population (9).

As observed in these findings, we concluded that agenesis is the most prevalent dental anomaly found in NSCL±P patients inside the cleft area and it is associated to wide-ranged sub phenotypes. Other disorders of number, size, eruption and location were also observed. However, as oral clefts have a multi factorial etiology, we should not ignore that genetic and environmental factors may influence dental anomalies development, even inside the cleft area. Our results demonstrate that dental anomalies should be considered during dental treatment planning of individuals affected by NSCL±P.
